# Neuronal Population Transitions Across a Quiescent-to-Active Frontier and Bifurcation

**DOI:** 10.3389/fphys.2022.840546

**Published:** 2022-02-10

**Authors:** Drandreb Earl O. Juanico

**Affiliations:** ^1^DataSc/ense TechnoCoRe, Technological Institute of the Philippines, Quezon City, Philippines; ^2^NICER Program, Center for Advanced Batteries, Quezon City, Philippines

**Keywords:** neuronal avalanches, differential inclusion, excitation-inhibition balance, epileptiform activity, homeostatic regulation

## Abstract

The mechanistic understanding of why neuronal population activity hovers on criticality remains unresolved despite the availability of experimental results. Without a coherent mathematical framework, the presence of power-law scaling is not straightforward to reconcile with findings implying epileptiform activity. Although multiple pictures have been proposed to relate the power-law scaling of avalanche statistics to phase transitions, the existence of a phase boundary in parameter space is until now an assumption. Herein, a framework based on differential inclusions, which departs from approaches constructed from differential equations, is shown to offer an adequate consolidation of evidences apparently connected to criticality and those linked to hyperexcitability. Through this framework, the phase boundary is elucidated in a parameter space spanned by variables representing levels of excitation and inhibition in a neuronal network. The interpretation of neuronal populations based on this approach offers insights on the role of pharmacological and endocrinal signaling in the homeostatic regulation of neuronal population activity.

## Introduction

The debate on whether and how the brain operates at a critical state is far from settled (Muñoz, 2018; Wilting and Preisemann, 2019; Plenz et al., 2021). What is common among experimental observations of cortical network activity referred as neuronal avalanches is that the size of such events (measured in several ways) can span across extended length and time scales. Generating mechanisms have been proposed, and the scientific consensus is undecided. However, glaring and recurring facets of experimental evidence must be governed by a simple corroborating explanation.

Most of the mathematical approaches used to describe neuronal population dynamics are based on differential equations and thresholds. The differential equation offers a convenient method of analysis because of the rich mathematical tradition it holds. Through uniqueness and existence theorems, differential equations can produce reliable expressions that can be used to predict natural phenomena, such as neuronal population activity. However, this reliable method does not necessarily provide a complete and sufficient picture of the phenomena. For instance, the Gillespie stochastic simulation algorithm and chaos theory must add “roughness” to the otherwise smooth analytic results from differential equations to assimilate information from real-world data. Yet, another generalization of differential equations is the differential inclusion (Gast, 2020). The differential inclusion admits the possibility of multiple solutions that pass through a single initial value. This set-valued property of the differential inclusions is a reasonable starting point to convert the inherent discontinuities of individual neuron properties to the scale of neuronal populations.

The salient properties of neuronal avalanches observed in laboratory experiments offer the clues necessary to complete the picture. Although the power-law scaling of avalanche size and duration statistics received the broadest attention, there are additional results that deserve more notice. One of these results is the emergence of a bimodal distribution of avalanche sizes when the neuronal network is disinhibited in a certain way. This property can be transitory, such as those produced by pharmacological methods, after which the power-law scaling recovers (Plenz et al., 2021). However, this transient property could already hint at the elements that a more adequate mathematical picture of neuronal population activity must possess.

This paper offers a framework with which to make sense of the variety of properties observed in the neuronal avalanche statistics. A parameter space spanned by variables representing the levels of excitation and inhibition is mapped to elucidate the boundary between a quiescent and active state of the neuronal network. Through this framework, new insights are offered to explain the characteristics of neuronal population activity, such as in relation to the emergence of epileptiform episodes.

## Differential Inclusion Model

Neuronal population models are inherently discontinuous with respect to the dependent variable, assuming threshold activation. Although threshold activity is evident in single neurons, it is less clear how threshold activity translates to the level of population or networks. Various proposals to address this gap between microscopic timescale of spikes and macroscopic timescale of collective network behavior (Tkačik et al., 2015) include notions of synchronized firing and synfire chains (Plenz et al., 2021). Scaling assumptions may be taken to mean that discontinuous neurons also imply discontinuous neuronal populations. Thus, a discontinuous population model, specifically a differential inclusion model (DIM) with a discontinuous right-hand side, is suitable for describing the neuronal network dynamics. It is a straightforward way of translating cell-level discontinuity to the scale of a network.

The common mathematical models that have been used to describe the dynamics of neuronal activity are constructed from systems of differential equations. For instance, the celebrated mathematical models of FitzHugh and Nagumo, Hodgkin and Huxley, and others, such as the Wilson-Cowan equations (di Santo et al., 2018) are expressed as differential equations. The drawback with differential equations is that they presume the uniqueness of the solution to any initial-value problem, which requires that the equations must satisfy at least Lipschitz-continuity conditions. On the other hand, DIMs admit any feasible (under certain closure assumptions) solutions through every initial point (Benaïm et al., 2005). Gast (2020) showed that DIM solutions can be found numerically using stochastic simulations in such a way that stochastic trajectories converge to the set of solutions admitted by the DIM.

The interplay between inhibition and excitation is assumed to be behind the network activity patterns, but the explicit roles each take in this process is unclear. For instance, in some models, disinhibiting the neuron does not necessarily lead to a disinhibited network, i.e., displaying the bimodal activity observed in multi-electrode array experiments. This gap must be seriously considered. One of the commonly held assumptions is that neurons tend to contribute to the excitation of other neurons to which it makes direct electrophysiological connections, or be excited by the sum of inputs supplied by those incoming direct connections. However, it is well established in the neurophysiology literature that other non-neuronal cells, such as glial cells, also influence network activity (Zierenberg et al., 2018). Also, neurons have high membrane resistance that causes nerve connections to dissipate energy like memristors, as revealed by measurements based on impedance spectroscopy (Bou and Bisquert, 2021). This dissipation implies that nerve membranes do not achieve superconductivity for any amount of electric charge, which is consistent with the dissipative equivalent circuits deduced to represent neuronal electrical behavior.

Villegas et al. (2019) recognized the contact process as a suitable approach for the population-wide propagation of active potentials from neuron to neuron across the network. Unlike models of self-organized criticality (SOC), the assumption of infinite time-scale separation between external driving and dissipation is not necessary. Contact-process models are also seen as straightforward representation of spreading dynamics, as in the context of epidemics (Muñoz, 2018; Korchinski et al., 2021). Within the context of a contact process, it is easier to interpret the existence of two or more types of otherwise identical units, such as neurons, which can differ from one another at any given point in time by virtue of their membrane potentials that are either above or below a threshold.

**FIGURE 1:**
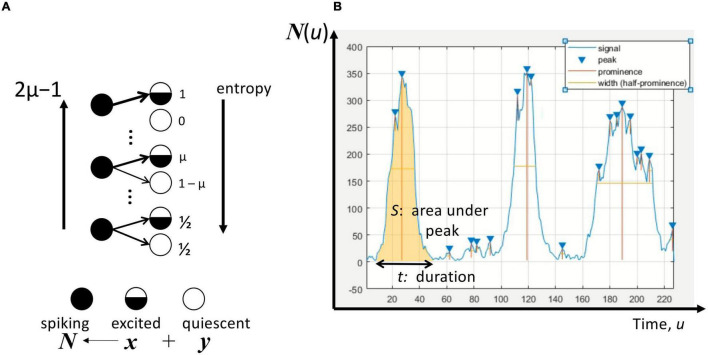
Avalanches in the neuronal population/network. **(A)** The excitation by a spiking neuron with a probability μ for an excited neuron, and 1-μ for a quiescent neuron, where μ∈[1/2,1]. The excitation parameter 2μ–1 is inversely related to the information entropy of this excitation process. At maximum entropy, μ = 1/2, a neuron’s subsequent excitation is not dependent on its state. At minimum entropy, μ = 1 implying that only excited neurons can be activated by a spiking neuron. A total of ***N*****(*****u*****)** spiking neurons at time ***u*** comprises ***x*** neurons that were at the excited state and ***y*** neurons at the quiescent state immediately beforehand. **(B)** The number of spiking neurons ***N*** for any given time ***u*** as a time-series signal. The peaks of this time series are recognized using Matlab’s *findpeaks* algorithm by setting the prominence to 1. The avalanche duration ***t*** is the estimate width between consecutive troughs, whereas the avalanche size ***S*** is the total area under the peak between the troughs.

Following Juanico (2014, 2015), the rate of excitation of a neuron depends on whether it is excited or quiescent. The probability per unit time that presently spiking neurons activate an excited (quiescent) neuron is μ (1-μ) as illustrated in Figure 1A. Thus, μ or, more precisely, 2μ–1 is the excitation parameter with values between 0 and 1. This parameter can take a similar meaning as the inverse of information entropy because at the value of 0 this entropy is maximum as the excited and quiescent neurons are no longer distinguishable. On the other hand, at a value of 1, the excitation parameter corresponds to a situation of minimum entropy. With this interpretation, 2μ–1 therefore becomes a structural parameter that allows us to explore how this assumption of connectivity influences the characteristics of population activity, addressing the gap of scaling the representation from individual neurons to neuronal networks.

Inhibition can be described on the local (i.e., connection-based) and global (e.g., pharmacological or endocrine/hormonal signaling) levels. Locally, an appropriate contact-process representation will be density-dependent, i.e., higher number of spiking neurons triggers stronger local inhibitory influences (Savin and Tkačik, 2017). Local inhibition influences the alternation of periods of activity and silence, proposed as an essential inhibitory control on neural encoding (di Santo et al., 2018). Globally, a diversity-based approach seems reasonable, especially at the level of populations, i.e., neuronal networks or neural mass (Fan et al., 2019; Deschle et al., 2021). Global inhibition such as endocrine signaling has not been explicitly considered in previous descriptions of neuronal network activity. For instance, *in vivo* networks appear “subcritical” (Preisemann et al., 2014), i.e., avalanche sizes do not span across extended scales, compared to *in vitro* (organotypic) samples (Zierenberg et al., 2018), possibly because of a higher degree of global inhibitory feedback exerted by the full homeostatic regulation at work within the organism (Lignani et al., 2020).

Consider a random network of ***N*** spiking neurons in which ***x*** had membrane potential at or near the threshold (i.e., “excited” neurons) and ***y*** had membrane potential below the threshold (Figure 1A). The membrane potential need not be the same across all neurons in the network at any given time, as emphasized by Deschle et al.’s (2021) critique of neural mass models. A random network structure is also a reasonable description of connectivity, whether it is directed (Deschle et al., 2021) or not (Liang et al., 2020). These elements of excitation, local and global inhibition combine additively to the following DIM:


$$\dot{\text{x}}\in \left[\mu -\delta \,\text{x}-\nu \,\Phi \,\left(x,y\right)\right]\,\text{N}$$

$$\dot{\text{y}}\in \left[1-\mu -\delta \,\text{y}-\nu \,\Phi \,\left(x,y\right)\right]\,\text{N}$$

With ***x*** + ***y*** = ***N*** the DIM can further be simplified into the following:


$$\dot{\text{N}}\in \left[1-\delta \,\text{N}-2\,\nu \,\Phi \,\left(x,y\right)\right]\,\text{N}$$

which reveals the three competing mechanisms: excitation represented by the first term; local density-dependent inhibition in which the parameter δ is inversely proportional to system size (i.e., δ^–1^ > ***N***); and global inhibition by the third term, which contains the inhibition parameter ν and the set-valued function Φ, which can be written as,


$$\Phi \,\left(x,y\right)=\left\{ \begin{array}{cc} 0 & 0<x,y\leq 1\\ \frac{x\,\left(x-1\right)+y\,\left(y-1\right)}{\left(x+y\right)\,\left(x+y-1\right)} & x,y>1\\ 1 & 0<x||\text{y}\leq 1,y||x>1 \end{array}\right.$$

This piecewise definition of Φ indicates that it takes one spiking neuron (i.e., the threshold value for ***N***) to trigger a neuronal avalanche, which is the essential piece of the recipe in SOC models. At the limit of large values of ***x*** and ***y***, this set-valued function is equivalent to the Simpson diversity index. One consequence of the discontinuity inherent in Φ is that a pure population of spiking neurons comprising only the formerly excited ones drives an inhibitory feedback twice stronger than driving a perfectly mixed spiking population consisting of equal proportions of formerly excited and quiescent neurons.

Using the contact process as the framework, the avalanche dynamics can be analyzed from the solutions of the DIM. The neuronal avalanche is the event representing the spiking activity of multiple neurons at a specific point in time. The number of spiking neurons is denoted by ***N***, and from this number’s evolution in time the size and duration of the avalanche can be estimated (Figure 1B).

Solutions of the DIM were obtained using Gillespie stochastic simulation. Stochastic simulation also provides the randomness needed for exploring the concept of statistical criticality (Muñoz, 2018; Wilting and Preisemann, 2019). Stochastic dynamics is indeed known to be an essential property of neuronal network activity, as previous studies established.

## Materials and Methods

### Gillespie Stochastic Algorithm

**FIGURE 2:**
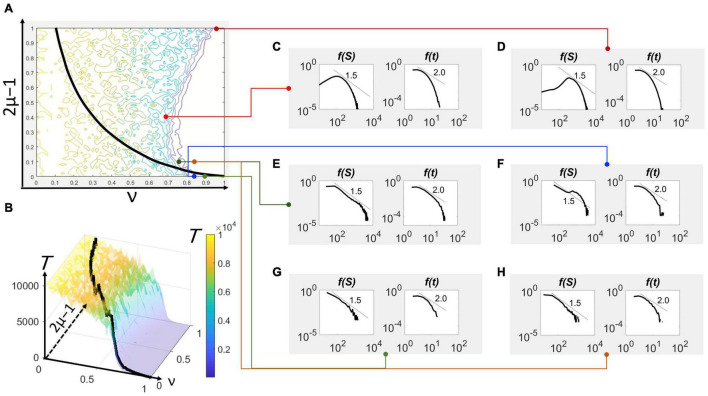
Avalanche size and duration statistics in parameter space. **(A)** The contour plot of the extinction time with respect to the excitation parameter, 2μ–1, and global inhibition parameter, ν, for δ = 0.0005 (or δ^– 1^ = 2,000). Superimposed on the contour plot is the bifurcation locus (black curve). **(B)** The three-dimensional view of the contour also showing the same bifurcation locus (black curve). The color scale representing the extinction time ***T*** applies to the contour plot as well. The maximum value of ***T*** has been set to 10^4^ for faster computation, although it could have been set at a higher order of magnitude. Avalanche size (left) and duration (right) histograms, shown in double logarithmic scale, from simulations of the model with specific parameters mapped in **(A)**. **(C)** 2μ–1 = 0.40, ν = 0.70. **(D)** 2μ–1 = 1.00, ν = 0.92. **(E)** 2μ–1 = 0.10, ν = 0.75. **(F)** 2μ–1 = 0.00, ν = 0.85. **(G)** 2μ–1 = 0.00, ν = 0.90. **(H)** 2μ–1 = 0.10, ν = 0.85.

The direct method was used to find feasible solutions to the DIM from an initial condition of a single spiking neuron emerging randomly from an excited or a quiescent neuron, i.e., ***N***(0) = 1. For every stochastic realization, the extinction was recorded at the time ***T*** wherein ***N***(***T***) = 0. The ensemble average of ***T*** was calculated for every given combination of the excitation and global inhibition parameters at a given system size/local inhibition parameter, δ. The values are visualized in a contour plot (Figure 2A) and surface plot (Figure 2B). The number of realizations to obtain sufficient convergence can vary depending on the variability across stochastic results. Fewer realizations were needed if this variability is low, whereas more were considered for high variability.

The DIM was also solved numerically using Matlab *ode45*, a numerical solver based on the Runge-Kutta method with adaptive step size for efficient computation. Although the results from this numerical computation do not necessarily represent the mean field, stochastic convergence can be shown for some parameter combinations.

### Extinction Time

The extinction time ***T*** is the solution to the boundary value problem derived from the backward Fokker-Planck representation of an autonomous process that takes the trajectory of ***x*** to 0. If the DIM will tend to saturate to a non-zero value of ***N***, then the expected ***T*** will tend to infinity, corresponding to the stable dynamics within the context of avalanche criticality (Wilting and Preisemann, 2019). However, infinite activity is also not physically reasonable. Sustaining electrical signal propagation with a single pulse of input, the neuronal network’s connections must become superconductive (Bou and Bisquert, 2021), which is inconsistent with the physiologically supported notion of a dissipative neuron. For this reason, an avalanche is unlikely to have an unbounded average size, which is equivalent to stating that avalanches are not likely to last indefinitely. Inhibition is a necessary feature that naturally silences neuronal population activity, preventing it from over-activating the system and damaging the organism. It is interesting to find conditions for the existence of finite expected values of ***T***. Consider the equilibrium ***x*** **=** x* and a parameter *K* that tends to infinity as the network size expands. Then,


$$\text{T}\,\left({\text{x}}^{*}\right)=2\,\text{K}\,\int_{0}^{x^{*}}\int_{r}^{\infty }\frac{\text{Exp}\,\left[2\,\text{K}\,\int_{r}^{s}\frac{\mu -z-\nu \,\Phi \,\left(z\right)}{\mu +z+\nu \,\Phi \,\left(z\right)}\,\text{dz}\right]}{\left(\mu -\text{z}-\nu \,\Phi \,\left(s\right)\right)}\,\text{dsdr}$$

The term inside the square brackets must be less than zero so that ***T*** will be finite. Close to extinction, it is expected that the number ***y*** of neurons that were quiescent before triggered to spike is much lower than the number ***x*** that were already excited. Also, the ratio ***y***/***x*** can be estimated as a function of the excitation parameter. From the Taylor expansion of Φ,


$$\frac{\text{y}}{\text{x}}\approx {\left(1-\mu \right)}^{2}\,\text{A}$$

in which ***A*** is a constant. From this approximation, a curve representing a constraint for 2μ–1 and ν can be expressed. This curve appreciably fits (not shown) the shape of the frontier (Figure 2A).

### Bifurcation Locus

The DIM has a hidden transcritical bifurcation satisfied by the locus defined by the following constraint (Juanico, 2014):


$$2\,\mu -1=\frac{2}{3}\,\sqrt{\frac{{\left(1-\nu \right)}^{3}}{3\,\nu }}$$

As ν→1, the locus degenerates to the point 2μ–1 = 0 (Figures 2A,B), which recovers the case of maximum information entropy, μ = 1/2 (Figure 1A), corresponding to the case wherein every neuron in the population is equally likely to spike regardless of its present excitation state.

### Avalanche Size and Duration

The Matlab subroutine named *findpeaks* was used to automate the identification, measurement, and location of the peaks in a time series signal. This algorithm iterates a nearest-neighbor comparison. The value of the peak detected and its horizontal width are two of the quantities that *findpeaks* generates efficiently. Following Villegas et al. (2019), the avalanche size is the area under the curve peaks, while the avalanche duration is estimated through the width between consecutive troughs. Unlike the approach by Villegas et al. (2019) however, the current implementation does not require a threshold value as ***N***, by its very definition, is never less than zero. This method of implementation eludes the conceptual difficulty and ambiguity caused by thresholding when counting or measuring neuronal avalanches (Villegas et al., 2019; Wilting and Preisemann, 2019).

## Results

The excitation-inhibition (“E/I”) balance (Lombardi et al., 2012; Lignani et al., 2020; Plenz et al., 2021), in the sense of a static equilibrium, takes shape as a non-linear frontier/boundary between two regimes in the parameter space—the (active) up-state and (quiescent) down-state. This categorization of network state was previously provided by Millman et al. (2010) in a model that attempted to make an explicit representation of excitation and inhibition from the equations governing neuronal membrane circuitry. Unlike analyses based on commonly appealing notions of criticality (Muñoz, 2018), the results here explicitly visualize this balance in the parameter space representing excitation (2μ–1) and inhibition (ν), shown as a “frontier” in Figure 2A. This balance is much more pronounced in three-dimensions, such as Figure 2B showing the ***T***-surface falling off like a cliff when moving from left to right and crossing this frontier. The bifurcation locus traversing this surface illustrates the substantial drop in value. The maximum value of the plateau is only limited by the length of the observation period. On the other hand, the height of the valley does not change much despite lengthening this period.

The avalanche statistics provide the visual test of the model’s generalization. At several points around the frontier (Figure 2A), the statistical distribution of avalanche size and duration vary from shapes indicating hyperexcitability (Figures 2C,D), marginally disinhibited (Figure 2F), critical (Figures 2E,H), and damped or “sub-critical” (Figure 2G). The epileptiform activity associated to hyperexcitable states is characterized by avalanche sizes that have a peak at high values although the duration statistics exhibit robust power-law scaling. A marginally disinhibited activity, on the other hand, displays the tail hump and a steeper fall off to the hump, as observed in previous laboratory studies of neuronal avalanches (Plenz et al., 2021). The critical behavior characterized by the exponent of 1.5 for avalanche sizes and 2.0 for duration seems to appear in the vicinity of both the frontier and bifurcation locus. Finally, subcritical behavior is well within the quiescent regime in the parameter space, in which the population activity initiated by a single spiking neuron dies out within a characteristic scale. This scale is apparent in both the avalanche size and duration statistics tapering off faster than the power law.

**FIGURE 3:**
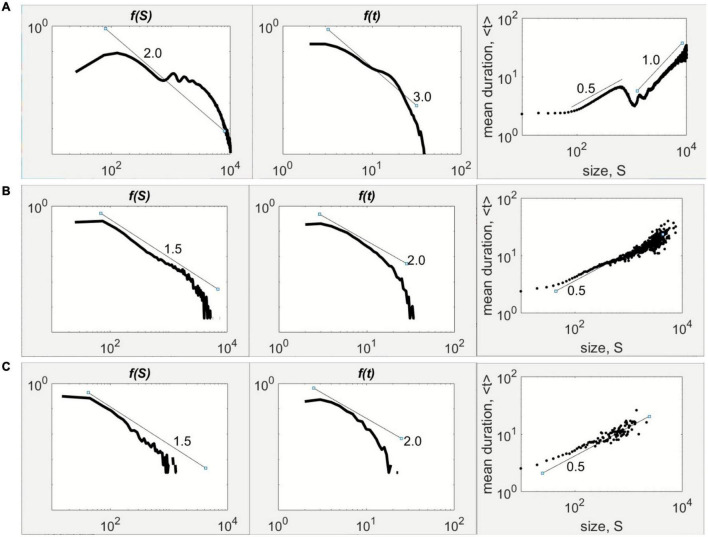
Avalanche statistics influenced by global inhibition. The magnitude of the global inhibition parameter ν can induce different shapes of the avalanche size, *f(S*), and duration statistics, *f(t*), in double logarithmic scale, for 2μ–1 = 0.10. **(A)** Bimodal shape of with a tail hump for *f(S*) and a scaling of exponent –2, while *f(t*) scales as –3 at low ν. The mean duration scales with size in two regimes characterized by two exponents separated by a gap. **(B)** The power-law of *f(S*) with exponent –3/2 at critical ν with the accompanying power-law of *f(t*) with exponent –2. The duration scales with size with a scaling exponent of 1/2. **(C)** Faster tail decline at high ν for both *f(S*) and *f(t*). The scaling between duration and size with an exponent of 1/2 occurs over a limited extent.

The change from supercritical through critical and subcritical regimes happens by crossing the frontier. For the case of 2μ–1 = 0.1, the supercritical (hyperexcited/epileptiform) behavior is seen when ν = 0.65, which is to the left of the frontier (Figure 2A). The size statistics display the bimodal shape with a peak at small sizes and another near the system size, although the duration statistics exhibits the expected power-law scaling (Figure 3A). At the frontier (2E), on which ν = 0.75, the statistics possess the well-known –3/2 and –2 power-laws for the avalanche size and duration, respectively (Figure 3B). Lastly, at ν = 0.85 (Figure 2H), which is to the right of the frontier and within the quiescent regime of the parameter space, the avalanche statistics developed the characteristic scaling indicating the constrained population spiking activity (Figure 3C).

For further validation, the transitions across the frontier were also examined through the scaling between the average avalanche duration and avalanche size, as presented in the rightmost column of Figure 3. In the supercritical case (Figure 3A), the duration and size are scattered in two scaling regimes separated by a gap. The first regime follows closely a scaling exponent of 1/2, whereas the other regime with exponent of 1. This scaling behavior associated with a hyperexcited neuronal population has been observed experimentally in folate-reared neuronal cultures (Yaghoubi et al., 2018). The critical case (Figure 3B) exhibited a 1/2-scaling law between duration and size, which is consistent with more elaborate statistical arguments outlined elsewhere (Shaukat and Thivierge, 2016). The subcritical case (Figure 3C) retains the 1/2-scaling to a limited extent due to the short lifetime of population activity in the regime to the right of the frontier (Figures 2A,H).

**FIGURE 4:**
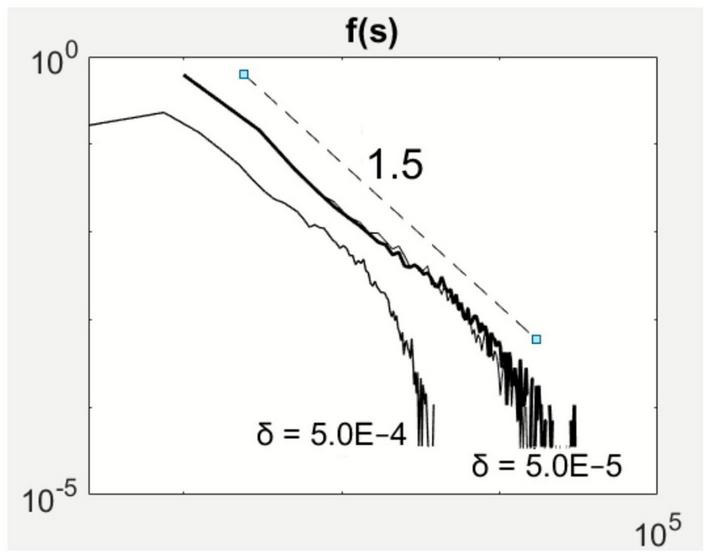
System size effects on avalanche size statistics. Reducing the parameter effectively increases the system size relating to local inhibition. The scaling exponent (1.5) appears to be robust to the change in system size.

The increase in system size by reducing δ does not affect the power-law scaling in avalanche size (Figure 4). At 2μ–1 = 0.1 and ν = 0.75 in parameter space, the avalanche size distribution displays the –3/2 power-law scaling (Figures 2E, 3B). By reducing δ by an order of magnitude, the scaling law spanned a broader extent while maintaining the –3/2 exponent. However, to observe this extension also requires longer observation periods as large sizes occur at much smaller probabilities.

**FIGURE 5:**
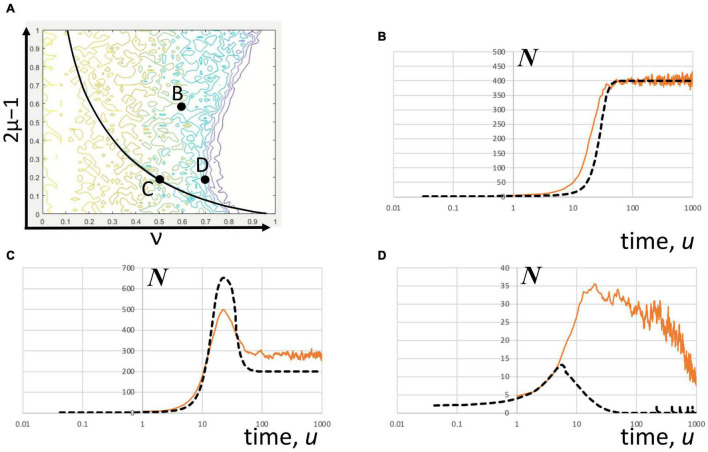
Convergence of stochastic solutions of the DIM. The Gillespie method covers admissible solutions that match the “mean-field” graph for different excitation and global inhibition parameters. **(A)** The parameter map showing the indicating parameter pairs. The stochastic solution is the average of multiple stochastic realizations (orange) and the “mean-field” solution (black dash) was estimated by numerically solving the DIM equations at the initial condition, ***x*** = 1 and ***y*** = 1. The color scale is the same as shown in Figure 2B. The number of realizations considered varied in rough proportion to the variance between realization. **(B)** Far from the frontier (2μ–1 = 0.6, ν = 0.6; number of realizations = 20) the stochastic solution converges to a high level of sustained number of spikes agreeing with the mean field solution. **(C)** Also in the interior and on the bifurcation locus (2μ–1 = 0.2, ν = 0.5; number of realizations = 84), the averaged stochastic trajectories initially ramp up and peak at a certain time before it declines and stabilizes. The peak predicted is shorter than the estimated mean field solution while the stabilization value is higher. **(D)** Near the frontier (2μ–1 = 0.2, ν = 0.7; number of realizations = 105), the stochastic trajectories include sustained oscillations that extend the extinction time of the population activity, which explains why the stochastic average of these trajectories substantially overshoot the peak predicted by the mean-field estimates.

The stochastic realizations of the DIM corresponding to different points in parameter space (Figure 5A) do not strictly converge to a mean-field solution obtained by numerical integration. The stochastic convergence seems to depend on whether or not the parameters are found near or within the frontier. Inside the frontier, the stochastic realizations show hyperexcited state of the network with sustained population activity at a high level (Figure 5B). At this level of excitation (i.e., 2μ–1 = 0.6), the neuronal network stabilizes to a maximum level of activity. Also, inside the frontier, but on the bifurcation locus, the activity peaks, on average, before it stabilizes to a level lower than the peak (Figure 5C). Near the frontier, however, the deviation of the stochastic average from the mean field is more pronounced. The average peak activity is significantly higher and occurs later than predicted by the numerical solution of the DIM for this case (Figure 5D). These observations apparently corroborate the findings of multiple solutions above criticality by Allegrini et al. (2010).

## Discussion

The mathematical structure of the DIM proposed herein must subscribe to the assumption that the neuronal state is a quantum state, specifically a qubit. This quantum picture is hinted by the notion of “diversified elements” in the work of Allegrini et al. (2010). Indeed, in the words of Koch and Hepp (2006), a neuronal network inside a biological organism “must obey the laws of physics, both classical and quantum.” However, the conceptual difficulties of reconciling neurophysiological knowledge about how neurons exchange information and the requisite of coherence for quantum states persist because the definition of a “neuronal quantum state” has been unclear. The present study postulates that the excitability level (i.e., how close the membrane potential is to the threshold) can be interpreted as a qubit, which explains why the DIM interprets a spiking neuron at a certain time as being previously an excited or quiescent neuron. The excitation arising from a stimulus originating from one neuron can be regarded as a form of quantum measurement in the sense recently formalized by Khrennikov and Asano (2020). Sustaining the coherence of this qubit to allow the simultaneous existence of the two states until a quantum measurement event takes place in a neuron may be possible because of background activity or spontaneous neuronal noise (Juanico and Monterola, 2007). Indeed, coherent spontaneous fluctuations in neuronal activity have been suggested as a possible explanation of the variability in human behavioral response found in controlled experiments (Fox et al., 2006). Consequently, by admitting the qubit nature of the neuronal state, its excitability level at any given point in time, leads to the mathematical parsimony of the DIM.

Persistent phenomena must indeed emerge from simple explanations, and for neuronal population activity, no phenomenon could be more persistent than those suggesting an E/I balance operating in a neuronal system. Several attempts have indeed been proposed to elucidate the notion of E/I balance (Deschle et al., 2021), even recently finding its way to applications for the clinical detection of epileptic seizure or epileptogenesis (Fan et al., 2019; Lignani et al., 2020). This attempt to clarify the meaning of E/I balance is even less apparent in previously proposed representation of the phenomena, e.g., critical branching process, Boltzmann’s chaos (Touboul and Destexhe, 2017), neutral theory (Martinello et al., 2017), directed random network of integrate-and-fire neurons (Millman et al., 2010). For instance, Millman et al. (2010) proposed to change the ratio between the inhibitory and excitatory currents to shift the network’s state between quiescent and active regimes. However, even with their straightforward network-of-neurons approach, the model by Millman et al. (2010) did not generate a state of hyperexcitability that is associated to the observed bimodal shapes of the avalanche statistics (Plenz et al., 2021). Lombardi et al. (2012) and Lignani et al. (2020) previously offered an interpretation of E/I balance within the context of homeostatic regulation, an involuntary mechanism in the organism that can drive the neuronal network to swing between up and down-states. The unclear aspect of this interpretation, however, is the definition of the quiescent-to-active boundary that the system’s state crosses over and how this crossover can take place. Notwithstanding, even a proposal to view criticality based on synchronization phase transition instead of a quiescent-to-active transition (di Santo et al., 2018) falls short of capturing the qualitative change in the avalanche size distribution when the network is disinhibited intentionally (e.g., organotypic cortex cultures) or by virtue of its incomplete structural development (e.g., dissociated cultures).

This paper sought to map the parameter space to elucidate the quiescent-to-active boundary that is the basis of the phase transition. Some previous work also used a parameter mapping approach to elaborate the shortcomings of mean-field based frameworks. For example, Deschle et al. (2021) showed that integrate-and-fire neural mass models, which originated as far back as Brunel’s (2000) canonical work, predict population activity only within a small region of a parameter space. To this end, the DIM can be interpreted as probing the parameter space of possible models, according to Tkačik et al. (2015), who proposed the entropy-based framework of criticality. Indeed, the MaxEnt framework is consistent with finding an optimal solution in the set spanned by a differential inclusion. The DIM helped in this mapping because it admits multiple solutions that passes through a given initial value, unlike ordinary differential equation systems that require a unique trajectory for every given initial value. The multiple solutions for a single initial value is akin to the notion of chaos, which Touboul and Destexhe (2017) observed in the synchronized irregular regime in which neuronal population activity spreads throughout the network while triggering massive inhibition that silences the network. This tug-of-war between excitation and inhibition is also the statement about population activity expressed by the DIM. Indeed, as DIM clearly embeds the concepts of entropy and chaos, it is an approach that can be aligned with the proposal of statistical criticality (Muñoz, 2018; Wilting and Preisemann, 2019).

The DIM approach gives rise to a variety of avalanche statistics, which views the power-law scaling properties as a special case rather than a universal property. Non-universality is at odds with proposals that the neuronal avalanche process belongs to the same class as critical branching processes. On the one hand, this interpretation seems to support the non-critical mechanisms of generating persistent power-law scaling, such as Boltzmann’s molecular chaos (Touboul and Destexhe, 2017). On the other hand, the results from the DIM analysis suggest that a quiescent-to-active transition exists in parameter space spanned by excitation and inhibition variables. The visualization of this phase boundary provides a clearer interpretation why neuronal avalanche experiments report a variety of consistent findings beyond the power-law scaling of size and duration statistics.

The presence of a hyperexcited state is perhaps the most perplexing piece of evidence beyond the power-law scaling as it appears in both organotypic and dissociated cortical cultures due to possibly different explanations. The bimodal shapes of the avalanche size distribution seem to corroborate with findings for disinhibited organotypic cortex cultures and dissociated cortical cultures (Plenz et al., 2021), or the observation of “dragon king avalanches” (Costa et al., 2017; Kinouchi et al., 2019), or “system size events” (Yaghoubi et al., 2018). For organotypic cortex cultures, the pharmacological intervention (e.g., bathing with picrotoxin) likely reduces temporarily the influence of global inhibition, allowing the parameter ν to make a brief lateral excursion to the left of the frontier. On the other hand, for dissociated cultures, the global inhibition feature could be less fully developed, maintaining the value of ν to the left of the frontier. The bimodal statistics corresponding to marginally disinhibited cases are not seen in models that generated the same critical scaling with exponents –3/2 and –2 for avalanche size and duration, respectively.

The hyperexcited states appear at low entropy levels (i.e., high 2μ–1) implying that structural assumptions about cortical networks are at odds with notions of criticality based on power-law scaling of avalanche statistics. This structural assumption is inherent in branching processes, which assert that the propagation of activity is only forward and never in loops. Another structural assumption relates to the threshold-based activity of a neuron, essential to electrophysiology-inspired descriptions such as the integrate-and-fire model. Such models ascribe an assumed preference to excite neurons that are already at or above the threshold. The results generated by the DIM suggests that these assumptions about the neuronal activity propagation generates supercritical, instead of critical, neuronal networks. Experiments on neuronal cultures reared with folate implied the same selectivity of excitation that generate hyperexcited, system-size events (Yaghoubi et al., 2018). In that study, the scaling between the mean avalanche duration and sizes appear to be governed by two regimes separated by an apparent gap and characterized by different scaling exponents (1/2 and 1). Also the scaling part of the size statistics and duration appear to be governed by scaling exponents different from those predicted by branching processes and similar mean-field approaches that rely on differential equations. On the contrary, critical networks operate at high entropy levels, specifically where the bifurcation locus and frontier are intertwined (Figure 2A). At high entropy, the activity propagates more indiscriminately, without a dominating preference for one type.

The DIM also shows a non-trivial relationship between the average of multiple stochastic realizations and the mean-field solution obtained numerically. For instance, near or at the frontier, the delay in the average peak is indicative of avalanche-like activity, which could propagate across a wider portion of the network due to the intermittent synchrony. For instance, Touboul and Destexhe (2017) established the occurrence of waves of synchronization in a network state they described as “synchronous irregular,” which is proposed to be the outcome of molecular chaos. In this regime, inhibition dominates over excitation, which is consistent with the episodes of low activity between high activity, akin to a wave or oscillation. These waves likely resemble the properties of a system undergoing synchronization phase transition (di Santo et al., 2018).

Finally, it would also be interesting to see how the parsimonious DIM approach can make sense of other systems that exhibit intermittent synchronization. For example, the recurrent surges of epidemic cases resemble the population dynamics arising from neuronal avalanches. Here, excitation is analogous to infection, while the susceptibility of any individual can be taken as a qubit. Epidemic spread is also a contact process with analogous elements of local inhibition (e.g., local healthcare facilities with finite capacity) and global inhibition (e.g., public health policy and interventions). Still, other systems, which have been mathematically described by differential equations, may benefit from a DIM representation through simplified expression and interpretation, yet richer dynamical outcomes.

## Data Availability Statement

The original contributions presented in the study are included in the article, further inquiries can be directed to the corresponding author/s.

## Author Contributions

The author confirms being the sole contributor of this work and has approved it for publication.

## Conflict of Interest

The author declares that the research was conducted in the absence of any commercial or financial relationships that could be construed as a potential conflict of interest.

## Publisher’s Note

All claims expressed in this article are solely those of the authors and do not necessarily represent those of their affiliated organizations, or those of the publisher, the editors and the reviewers. Any product that may be evaluated in this article, or claim that may be made by its manufacturer, is not guaranteed or endorsed by the publisher.
